# Flow diverter treatment for MCA M1 aneurysms: clinical outcomes and predictors of occlusion

**DOI:** 10.3389/fneur.2026.1782648

**Published:** 2026-04-02

**Authors:** Zeran Yu, Jiabin Su, Xinjie Gao, Ruiyuan Weng, Yuchao Fei, Heng Yang, Yuxiang Gu, Wei Ni

**Affiliations:** 1 Department of Neurosurgery, Huashan Hospital of Fudan University, Shanghai, China; 2 National Center for Neurological Disorders, Huashan Hospital, Shanghai Medical College, Fudan University, Shanghai, China; 3 Department of Neurosurgery, The Affiliated Hospital of Yunnan University, The Second People’s Hospital of Yunnan, Yunnan University, Kunming, China; 4 The Stroke Center, The Affiliated Hospital of Yunnan University, The Second People’s Hospital of Yunnan, Yunnan University, Kunming, China; 5 Department of Neurosurgery, The First Affiliated Hospital of Fujian Medical University, Fuzhou, Fujian, China

**Keywords:** aneurysm occlusion, cerebral middle artery, endovascular treatment, flow diverter devices, intracranial aneurysm, ischemic complication

## Abstract

**Background:**

The role of flow diverters (FDs) in middle cerebral artery (MCA) M1 aneurysms is not well established. We assessed their safety, efficacy, and predictors of incomplete occlusion.

**Methods:**

Consecutive MCA M1 aneurysms treated with FDs (January 2022–May 2024) were retrospectively reviewed. Clinical, imaging, and procedural variables were analyzed for associations with ischemic complications and angiographic outcomes.

**Results:**

Fifty-eight patients (mean age 57.5 years; 46.6% female) underwent treatment with Pipeline (56.9%), Tubridge (20.7%), Surpass Evolve (12.1%), or Lattice (10.3%) devices. Postprocedural cerebral ischemia occurred in 25.9% (15/58), significantly more often in ruptured versus unruptured aneurysms (26.7% vs. 2.3%, *p* = 0.013). Rupture independently predicted ischemia (OR 15.273, 95% CI 1.547–150.765; *p* = 0.020). At a median follow-up of 13 months, complete occlusion was achieved in 77.4% (41/53). Aneurysm wall enhancement on MRI independently predicted incomplete occlusion (OR 6.566, 95% CI 1.395–30.903; *p* = 0.017).

**Conclusion:**

FDs may be an option for carefully selected MCA M1 aneurysms. Ruptured aneurysms are associated with a markedly higher risk of postoperative ischemic complications, and aneurysm wall enhancement on preoperative MRI independently predicts incomplete aneurysm occlusion after FDs treatment.

## Introduction

Middle cerebral artery (MCA) aneurysms, located distally from the circle of Willis, often exhibit wide-necked saccular or fusiform morphologies and are commonly regarded as a distinct and challenging subtype of intracranial aneurysms. In recent years, increasing use of flow diverters (FDs) for the management of complex intracranial aneurysms, including those involving the MCA, has attracted increasing clinical interest. Owing to the high metal coverage of FDs and their endoluminal therapeutic mechanism, patients require standardized dual antiplatelet therapy. As a result, aneurysm healing dynamics following FDs treatment and the risk of ischemic complications related to perforator occlusion have become areas of significant clinical concern and ongoing investigation (1, 2). Considering the potentially devastating consequences of blocking the branches of MCA M1, such as the lenticulostriate artery (3), the number of MCA M1 aneurysms treated with FDs is limited.

In this study, we retrospectively reviewed consecutive cases of patients with MCA M1 aneurysms treated with FDs. We aimed to explore the safety and effectiveness of FDs in the treatment of MCA M1 aneurysms.

## Methods

### Study patients

This retrospective, single-center cohort study enrolled consecutive patients with side-wall aneurysms of the middle cerebral artery (M1 segment) treated with flow diverter devices between January 2022 and May 2024. Inclusion required a diagnosis of an MCA M1 aneurysm confirmed using digital subtraction angiography (DSA). Both ruptured and unruptured aneurysms were included in the study.

Demographic data, aneurysm characteristics, and treatment details were collected systematically. Aneurysm characteristics, including location, morphology, maximum diameter, neck size, and post-treatment imaging outcomes, were assessed using DSA. The treatment details included the use of adjunctive coils and the type and size of the deployed FDs. Perioperative complications (occurring within 30 days) and delayed complications (occurring after 30 days) were identified based on imaging findings and clinical symptoms.

### Aneurysm wall imaging type and analysis

Vessel wall imaging (VWI) on MRI was performed on a 3.0 T Discovery MR750W (GE Healthcare) with a 32-channel head coil. Aneurysm wall enhancement (AWE) was defined as CR_wall > 1; Positive remodeling, remodeling index ≥ 1.05 (negative if < 1.05); And stenosis, ≤ 30% or > 30%. Intraluminal thrombus was noted as present/absent, with enhancement defined as CR_thrombus > 0. Disagreements were resolved by consensus.

### Treatment strategy

In our center, flow diversion for M1 aneurysms was considered in selected cases in which conventional treatment strategies were considered suboptimal or technically challenging. These situations included fusiform aneurysms, wide-neck aneurysms unsuitable for coiling or stent-assisted coiling, recurrent aneurysms after previous endovascular treatment, or lesions in which microsurgical clipping was considered high risk due to anatomical complexity. FDs were considered for ruptured MCA M1 aneurysms when clipping or coiling were not feasible because of unfavorable aneurysm morphology or high procedural risk.

### Endovascular procedures

Four types of FDs are available at our center: Pipeline Flex (Medtronic, USA), Tubridge (MicroPort, China), Surpass Evolve (Stryker, USA), and Lattice (AccuMedical, China). Pipeline FDs are composed of 48 braided wires, including 36 cobalt-chromium and 12 platinum wires. Tubridge FDs, approved by the National Medical Products Administration (NMPA) of China, are made of a nickel-titanium alloy and feature a flared end, facilitating their deployment in intracranial aneurysm treatment (4). Surpass Evolve devices is a self-expanding FDs braided from 64 wires consisting of cobalt-nickel-chromium and platinum-tungsten alloys (5). Lattice FDs, approved for clinical use in 2022, is a cobalt-chromium flow diverter equipped with a mechanical balloon delivery system that actively assists in stent expansion (6).

Patients undergoing treatment received dual anti-platelet therapy, typically consisting of aspirin (100 mg daily) and clopidogrel (75 mg daily), which was initiated at least 3 days prior to the procedure. Platelet function tests were routinely performed. In cases of clopidogrel non-responsiveness, aspirin (100 mg daily) was combined with ticagrelor (90 mg twice daily). For patients with ruptured aneurysms and insufficient anti-platelet preparation, intraprocedural anti-platelet therapy with body weight-adjusted tirofiban infusion (0.1 μg/kg/min) was initiated during the procedure and continued for 24 h postoperatively.

All procedures were performed under general anesthesia. After right femoral 6F sheath insertion, patients received heparin (100 U/kg). A guiding/intermediate catheter was positioned in the cavernous segment of the internal carotid artery, and a microcatheter navigated to the aneurysm for FDs deployment with full neck coverage. During the procedure, a weight-adjusted intravenous bolus of tirofiban (0.4 μg/kg/min) is administered immediately before FDs deployment, followed by a 24-h continuous infusion (0.1 μg/kg/min). Adjunctive coil embolization was performed within the aneurysm sac for large, wide-necked, or fusiform aneurysms. In patients with multiple aneurysms involving different vascular territories, the remaining lesions were treated in a staged manner at later sessions.

### Complication and management

Postprocedural imaging, including computed tomography (CT) and/or magnetic resonance imaging (MRI), were routinely performed at our center to evaluate ischemic or hemorrhagic complications. Ischemic strokes confined to the vascular territory of the treated vessel were recorded as ischemic events, whereas the presence of postprocedural blood on imaging was classified as a hemorrhagic complication. Transient neurological deficit was defined as temporary focal neurological deficit with no corresponding infarction on neuroimaging.

### Follow-up and outcome

The modified Rankin Scale (mRS) was used to assess clinical outcomes. Patients were followed up through telephone interviews or clinical visits at 6 months and 1 year after the procedure. Computed tomography angiography (CTA) or magnetic resonance angiography (MRA) was performed at 6 months, whereas DSA was recommended at 12 months post-procedure.

### Statistical analysis

Statistical analyses were performed using SPSS 30.0 (IBM). Continuous variables were presented as means ± standard deviations for normally distributed data or medians with interquartile ranges (IQR) for skewed distributions. Categorical data were compared using the chi-square test, and continuous data with the Mann–Whitney U test. Univariate logistic regression was used to assess baseline predictors of procedure-related complications and aneurysm occlusion. Statistical significance was set at *p* < 0.05.

## Results

### Patient and aneurysm characteristics

A total of 58 patients (mean age 57.53 ± 11.76 years; 46.6% female) underwent flow diverter implantation for MCA aneurysms at our center between January 2022 and May 2024 (Table 1). Five patients presented with subarachnoid hemorrhage secondary to aneurysm rupture. Aneurysm laterality was as follows: 17 (29.3%) were in the left MCA, 41 (70.7%) in the right MCA. The prevalence of comorbidities included hypertension (44.8%), diabetes mellitus (12.1%), smoking (15.5%), and alcohol consumption (29.3%). Among the treated aneurysms, 52 (89.7%) were saccular, and 4 (6.9%) were fusiform.

**Table 1:** Baseline characteristics.

Parameters	Patients (Total = 58)
Female	27 (46.6%)
Age, years, Mean, SD	57.53 ± 11.76
Medical history	
Hypertension	26 (44.8%)
Diabetes	7 (12.1%)
Smoking	9 (15.5%)
Alcohol use	17 (29.3%)
Subarachnoid hemorrhage	5 (8.6%)
Stroke (excluding SAH)	15 (25.9%)
Multiple aneurysms	
No	39 (67.2%)
Yes	19 (32.8%)
Maximal diameter of aneurysm, Mean, SD (mm)	7.93 ± 4.61
Neck diameter, Mean, SD (mm)	5.58 ± 3.17
Diameter of patent artery, Mean, SD (mm)	3.03 ± 0.81
Aneurysm side	
Left	17 (29.3%)
Right	41 (70.7%)
Aneurysm morphology	
Saccular	52 (89.7%)
Fusiform	4 (6.9%)
Recurrent intracranial aneurysm	4 (6.9%)

The values are represented as the number of patients (%). SD, standard deviation; IQR, interquartile range.

Four types of flow diverter devices were used: Pipeline in 33 patients (56.9%), Tubridge in 12 (20.7%), Surpass Evolve in 7 (12.1%), and Lattice in 6 (10.3%) (Table 2). Adjunctive coiling was performed in seven patients (12.1%), and one patient (1.7%) received an additional LEO stent due to incomplete aneurysm neck coverage. After stent deployment, jailed branches were observed in 51 patients (87.9%). Specifically, the lenticulostriate arteries were involved in 9 cases (17.6%), the M2 superior trunk in 4 (7.8%), the M2 inferior trunk in 1 (2.0%), the anterior temporal artery in 5 (9.8%), and the anterior cerebral artery in 1 (2.0%). Multiple branch involvement was noted in 31 cases (60.8%).

**Table 2:** Procedural characteristics and jailed branches following flow diverter treatment.

Parameters	Patients (Total = 58)
Type of FDs	
Pipeline	33 (56.9%)
Tubridge	12 (20.7%)
Surpass evolve	7 (12.1%)
Lattice	6 (10.3%)
Number of implanted FDs	
1	57 (98.3%)
2	1 (1.7%)
Adjunctive coiling	7 (12.1%)
Additional LEO stent	1 (1.7%)
Anti-platelet regimen	
Aspirin	1 (1.7%)
Aspirin + Clopidogrel	30 (51.7%)
Aspirin + Ticagrelor	24 (41.4%)
Aspirin + Rivaroxaban	1 (1.7%)
Clopidogrel + Ticagrelor	2 (3.4%)
Jailed branches	
None	7 (12.1%)
Lenticulostriate artery	9 (15.5%)
M2 superior trunk	4 (6.9%)
M2 inferior trunk	1 (1.7%)
Anterior temporal artery	5 (8.6%)
Anterior cerebral artery	1 (1.7%)
Jailed Branches Occlusion (Post-FDs Deployment)	Jailed = 51
Patent	44 (87.9%)
Reduced flow (still patent)	6 (10.3%)
Occluded	1 (1.7%)

The values are represented as the number of patients (%). SD, standard deviation; IQR, interquartile range.

All patients were maintained on anti-platelet therapy post-procedure: 30 (51.7%) received aspirin (100 mg daily) combined with clopidogrel (75 mg daily), 24 (41.4%) were administered aspirin (100 mg daily) plus ticagrelor (90 mg twice daily), one patient (1.7%) was prescribed aspirin (100 mg daily) with rivaroxaban (2.5 mg twice daily) due to venous thrombosis, another (1.7%) received clopidogrel (75 mg daily) and ticagrelor (45 mg twice daily) owing to aspirin intolerance, and one patient was maintained on aspirin (100 mg daily) monotherapy due to gastrointestinal bleeding.

**Figure 1:**
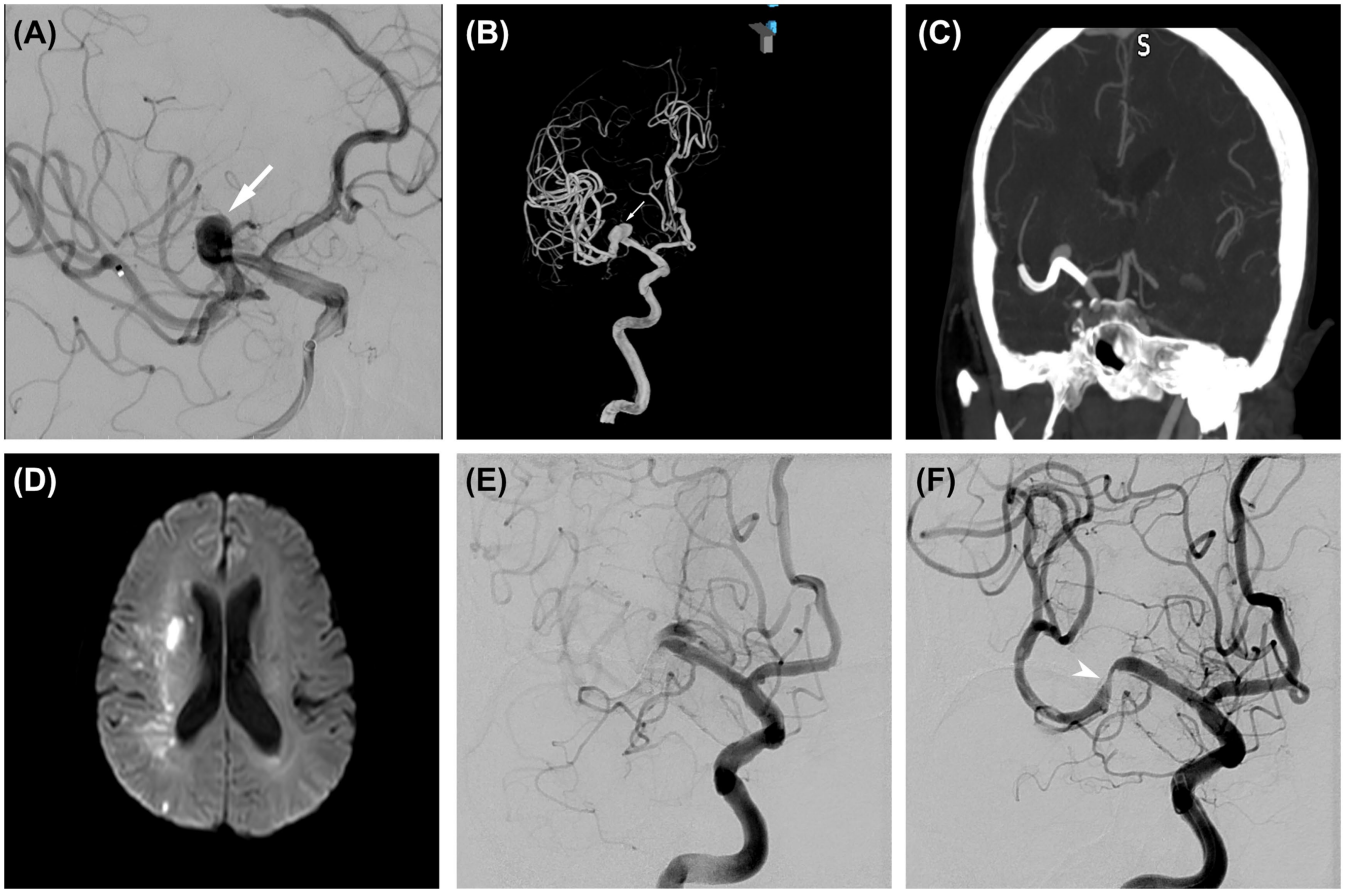
**(A)** Preoperative angiography showing a saccular aneurysm (arrow) in the M1 segment of the right middle cerebral artery. **(B)** 3D reconstruction of DSA showing the saccular aneurysm (arrow). **(C)** Postoperative computed tomography angiography showing that the flow diverter device completely covered the neck of the aneurysm and multiple branches. **(D)** On postoperative day 15, the patient developed sudden left limb weakness. DWI indicated an acute infarction in the right basal ganglia region. **(E)** Cerebral angiography showed slow blood flow through the stent and delayed opacification of distal vessels, suggesting in-stent thrombosis. **(F)** After thrombus aspiration and stent remodeling, blood flow through the stent improved, and the opacification time of distal vessels was reduced (arrow).

Among all treated patients, 4 (6.8%) experienced transient neurological deficit, 11 (19.1%) developed procedure-related cerebral ischemia/thromboembolism (Figure 1) and 2 (3.4%) had puncture site hematomas. No patient exhibited postoperative cerebral hemorrhage. Angiographic follow-up was performed in 53 patients who underwent FDs implantation. At the final angiographic assessment, 41 patients (77.4%) achieved complete aneurysm occlusion, whereas 12 (22.6%) showed incomplete occlusion (Table 3).

**Table 3:** Clinical and angiographic outcomes after flow diverter treatment.

Parameters	Patients (Total = 58)
Procedure-related complication	
None	41 (70.7%)
Transient neurological deficit	4 (6.8%)
Ischemic/thromboembolic	11 (19.1%)
Hemorrhagic	0
Others	2 (3.4%)
Last clinical outcomes with angiographic results (*n* = 53)	
Months after treatment, median (IQR)	13.0 (8.0–16.0)
mRS at last follow-up	
0–2	50 (94.3%)
3–5	3 (5.7%)
6	0
Aneurysm occlusion	
OKM A-B	12 (22.6%)
OKM C-D	41 (77.4%)

The values are represented as the number of patients (%). SD, standard deviation; IQR, Interquartile Range.

### Procedure-related ischemic complications after FDs implantation

Out of a total of 15 adult patients with cerebral ischemic events after the procedure (Table 4), 13 (86.7%) developed symptoms within 7 days, while two had delayed onset on days 10 and 14. Among infarctions, 3 (27.3%) resulted in severe strokes (mRS = 4), and 8 (72.7%) had minor infarcts with favorable outcomes (mRS ≤ 2). Device distribution in ischemic patients was Pipeline 7 (46.7%), Tubridge 1 (6.7%), Surpass Evolve 5 (33.3%), and Lattice 2 (13.3%). None of the patients required external ventricular drainage (EVD) during the acute phase of hemorrhage.

**Table 4:** Details of patients with procedure-related ischemic complications.

No.	Location and types of Intracranial aneurysm	Ruptured status, Hunt–Hess scale, and interval to treatment	Types of FDs	Jailed artery/Blood flow (post-FDs Deployment)^a,b^	Details of complications	Case description	Anti-platelet regimen	Timing of complications from procedure	Time of Follow-up (month)	Management and outcome
1	Saccular, L-M1	Ruptured, HHS = 1, 6 days	Tubridge (5.0*25)	LSA/1, ST/2	Right-sided motor impairment	Stent displacement with thrombosis formation,	Aspirin + Ticagrelor	3 days	6	Decompressive craniectomy, mRS = 4
2	Saccular, R-M1	Unruptured	Lattice (2.9*40)	LSA/1, ST/1	Left-sided motor impairment	In-stent thrombosis with ischemic infarction	Aspirin + Ticagrelor	10 days	6	Thrombus aspiration and Endovascular balloon angioplasty, mRS = 4
3	Saccular, R-M1	Unruptured	Pipeline (3*25)	LSA/1, ST/1	Left-sided motor impairment	Ischemic/thromboembolic	Aspirin + Ticagrelor	14 days	7	Conservative treatment, mRS = 1
4	Fusiform, R-M1	Unruptured	Pipeline (3*20)	ATA/1	Mild motor deficit	Transient neurological deficit	Aspirin + Clopidogrel	hours	8	Tirofiban, mRS = 0
5	Saccular, R-M1	Unruptured	Pipeline (2.75*20)	LSA/1, ST/1	Left-sided motor impairment	Ischemic/thromboembolic	Aspirin + Ticagrelor	hours	8	Supportive and symptomatic management, mRS = 2
6	Saccular, R-M1	Unruptured	Surpass Evolve (3.25*20)	ATA/1	Mild motor deficit with headache	Transient neurological deficit	Aspirin + Ticagrelor	3 days	18	Conservative treatment, mRS = 0
7	Saccular, R-M1	Unruptured, but recurrent after Clipping	Surpass Evolve (2.5*20)	ATA/1, IT/1	Mild motor deficit	Ischemic/thromboembolic	Aspirin + Ticagrelor	5 days	16	Comprehensive rehabilitation, mRS = 2
8	Saccular, R-M1	Ruptured after Clipping, HHS = 2, hours	Surpass Evolve (3.25*15)	ATA/1, ST/1	Altered consciousness with left-sided paresis	Ischemic/thromboembolic	Aspirin + Ticagrelor	2 days	15	Comprehensive rehabilitation, mRS = 4
9	Saccular, R-M1	Unruptured	Surpass Evolve (2.5*15)	ATA/1, IT/1	Mild motor deficit in left upper limb	Ischemic/thromboembolic	Aspirin + Ticagrelor	hours	24	Comprehensive rehabilitation, mRS = 1
10	Saccular, R-M1	Unruptured	Surpass Evolve (3.25*15)	LSA/1, ATA/1, ST/1	Mild motor deficit with headache	Transient neurological deficit	Aspirin + Ticagrelor	hours	18	Tirofiban, mRS = 0
11	Saccular, L-M1	Ruptured, HHS = 2, hours	Lattice (4.1*15)	ATA/1	Sensory aphasia	Ischemic/thromboembolic	Aspirin + Ticagrelor	3 days	15	Conservative treatment, mRS = 1
12	Saccular, L-M1	Ruptured, HHS = 2, 10 days	Pipeline (2.5*20)	LSA/1, ST/1	Mild motor deficit	Ischemic/thromboembolic	Aspirin + Clopidogrel	2 days	14	Conservative treatment, mRS = 1
13	Saccular, R-M1	Unruptured	Pipeline (3*20)	ATA/1, IT/1	Mild motor deficit	Ischemic/thromboembolic	Aspirin + Clopidogrel	hours	12	Tirofiban, mRS = 1
14	Saccular, R-M1	Unruptured	Pipeline (4*25)	ACA/1, IT/1	Mild motor deficit	Transient neurological deficit	Aspirin + Clopidogrel	hours	15	Tirofiban, mRS = 0
15	Saccular, R-M1	Unruptured	Pipeline (4*25)	ATA/2	Mild motor deficit	Ischemic/thromboembolic	Aspirin + Ticagrelor	hours	14	Tirofiban, mRS = 1

^a^LSA = Lenticulostriate artery; ST = M2 superior trunk; IT = M2 inferior trunk; ATA = anterior temporal artery; ACA = anterior cerebral artery. ^b^1 = Patent, 2 = Reduced flow (still patent), 3 = Occluded.

Transient neurological deficit was defined as clinically apparent deficits without corresponding ischemic lesions on MRI or CT.

Ischemic/thromboembolic was defined as new neurological deficits with corresponding ischemic lesions on MRI or CT.

HHS, Hunt and Hess Scale; mRS, modified Rankin Scale.

### Factors associated with procedure-related ischemia complications

Baseline variables potentially associated with procedure-related ischemic complications following FDs treatment are presented in Table 5. Factors analyzed included sex, age, medical history, aneurysm rupture status, aneurysm size, aneurysm wall enhancement on MRI, aneurysm morphology and laterality, presence of intraluminal thrombus, use of adjunctive coiling, and alloy composition of the FDs. Chi-square testing identified a significant association between ruptured aneurysms and procedure-related ischemia (*p* = 0.013). In concordance, univariate logistic regression demonstrated that aneurysm rupture was significantly associated with an increased risk of ischemic complications (OR = 15.273; 95% CI 1.547–150.765; *p* = 0.020). Although adjunctive coiling did not reach statistical significance, a higher rate of postoperative infarction was observed in patients treated with adjunctive coiling (26.7%, *p* = 0.066). Logistic regression analysis suggested a possible trend toward an increased risk (OR = 4.848; 95% CI 0.942–24.968; *p* = 0.059), indicating a potential association that may warrant further investigation in future studies. Additionally, clinical characteristics and outcomes at last follow up were compared between patients with ruptured and unruptured aneurysms (Table 6). Thrombosis within the aneurysm sac and left-sided aneurysms were more frequently observed in the ruptured aneurysm (80.0% vs. 22.6%, *p* = 0.018; 80.0% vs. 24.5%, *p* = 0.023, respectively). No significant differences were observed in aneurysm size, morphology, adjunctive coiling, and M2 trunk jailing. Among 53 patients with available clinical and angiographic follow-up data, patients with ruptured aneurysms had a lower rate of favorable functional outcome (mRS 0–2) than those with unruptured aneurysms (60.0% vs. 97.7%, *p* = 0.021).

**Table 5:** Predictors for procedure-related ischemia.

Parameters	Procedure-related ischemia			Univariate	
	Yes (*n* = 15)	No (*n* = 43)	*p* value	OR (95% CI)	*P* Value
Female	5 (33.3%)	22 (51.2%)	0.368	0.477 (0.140–1.631)	0.238
Age	59 ± 12	57 ± 12	0.539	1.018 (0.965–1.074)	0.506
Alcohol use	5 (33.3%)	12 (27.9%)	0.747	0.774 (0.219–2.793)	0.691
Smoking	3 (20.0%)	6 (14.0%)	0.682	1.542 (0.333–7.128)	0.580
Previous stroke	5 (33.3%)	10 (23.3%)	0.502	0.649 (0.140–2.999)	0.580
Ruptured aneurysm	4 (26.7%)	1 (2.3%)	0.013	15.273 (1.547–150.765)	0.020
Hypertension	4 (26.7%)	22 (51.2%)	0.136	0.347 (0.095–1.262)	0.108
Diabetes	2 (13.3%)	5 (11.6%)	1.000	1.169 (0.202–6.773)	0.862
Aneurysm size	6.69 ± 16.40	8.25 ± 24.33	0.399	0.934 (0.806–1.082)	0.369
Aneurysm wall enhancement			0.551	0.583 (0.176–1.928)	0.377
No	8 (53.3%)	17 (42.5%)			
Yes	7 (46.7%)	23 (57.5%)			
Thrombosis in aneurysm sac	4 (26.7%)	12 (27.9%)	1.000	0.939 (0.250–3.532)	0.939
Patent artery stenosis	3 (20.0%)	8 (19.0%)	1.000	1.062 (0.242–4.673)	0.936
Left aneurysm side	3 (20.0%)	13 (32.6%)	0.514	0.518 (0.126–2.136)	0.363
Aneurysm morphology			1.000	0.543 (0.058–5.062)	0.592
Saccular	14 (93.3%)	38 (88.4%)			
Fusiform	1 (6.7%)	5 (11.6%)			
Adjunctive coiling	4 (26.7%)	3 (7.0%)	0.066	4.848 (0.942–24.968)	0.059
Jailed branch	15 (100%)	36 (83.7%)	0.173	NA	
Any jailed M2 trunk	7 (46.7%)	25 (78.1%)	0.575	0.630 (0.193–2.053)	0.443
Alloy of FDs			0.156	4.812 (0.565–40.956)	0.150
Nickel-Titanium	1 (8.3%)	11 (91.7%)			
Cobalt Chromium	14 (30.4%)	32 (69.6%)			

OR, odds ratio; CI, confidence interval.

**Table 6:** Clinical characteristics and outcomes of ruptured and unruptured aneurysms.

Parameters	Ruptured aneurysm		*p* value
	Yes (*n* = 5) (8.6%)	No (*n* = 53) (91.4%)	
Recurrent intracranial aneurysm	1 (20.0%)	3 (5.7%)	0.310
Aneurysm size (mm)	10.65 ± 5.42	7.66 ± 4.62	0.171
Diameter of patent artery (mm)	2.98 ± 0.45	3.04 ± 0.84	0.936
Aneurysm wall enhancement	4 (80.0%)	27 (50.9%)	0.359
Thrombosis in aneurysm sac	4 (80.0%)	12 (22.6%)	0.018
Patent artery stenosis	2 (40.0%)	9 (17.0%)	0.237
Left aneurysm side	4 (80.0%)	13 (24.5%)	0.023
Aneurysm morphology			
Saccular	5 (100.0%)	47 (88.7%)	1
Fusiform	0	6 (11.3%)	
Adjunctive coiling	2 (40.0%)	5 (9.4%)	0.106
Any jailed M2 trunk	2 (40.0%)	30 (56.6%)	0.648
mRS 0–2^a^ (*n* = 53)	3/5 (60.0%)	47/48 (97.9%)	0.021

^a^Last clinical outcomes and angiographic results of 53 patients.

OR, odds ratio; CI, confidence interval.

### Factors associated with complete occlusion

Chi-square and logistic regression analyses were conducted to assess associations with incomplete aneurysm occlusion (Table 7). Although no statistically significant differences were observed in univariate analyses, trends were noted for diabetes (25.0%, *p* = 0.183) (OR 3.083, 95% CI 0.583–16.294, *p* = 0.185), aneurysm wall enhancement on MRI (75.0%, *p* = 0.042) (OR 4.235, 95% CI 0.951–17.999, *p* = 0.051), Nitinol alloy (33.3%, *p* = 0.150; OR: 0.343, 95% CI: 0.078–1.506, *p* = 0.156). Multivariate logistic regression revealed that aneurysm size trended toward an association with incomplete occlusion (OR 1.209, 95% CI: 0.991–1.475, *p* = 0.061), while aneurysm wall enhancement was significantly associated (OR 6.566, 95% CI 1.395–30.903, *p* = 0.017).

**Table 7:** Predictors for incomplete occlusion during follow-up.

Parameters	Incomplete Occlusion		*P* value	Univariate		Multivariate	
	Yes (*n* = 12) (22.6%)	No (*n* = 41) (77.4%)		OR (95% CI)	*P* Value	OR (95% CI)	*P* Value
Female	5 (41.7%)	19 (46.3%)	1.000	0.827 (0.225–3.039)	0.775		
Age	57 ± 11	58 ± 13	0.624	1.005 (0.954–1.059)	0.859		
Alcohol use	4 (33.3%)	12 (29.3%)	1.000	1.208 (0.305–4.783)	0.787		
Smoking	2 (16.7%)	5 (12.2%)	0.650	1.440 (0.242–8.567)	0.689		
Previous stroke	3 (25.0%)	10 (24.4%)	1.000	1.033 (0.233–4.578)	0.966		
Ruptured aneurysm	2 (16.7%)	3 (7.3%)	0.315	2.533 (0.371–17.280)	0.343		
Hypertension	6 (50.0%)	18 (43.9%)	0.751	1.278 (0.352–4.636)	0.709		
Diabetes	3 (25.0%)	4 (9.8%)	0.183	3.083 (0.583–16.294)	0.185		
Aneurysm size	6.30 ± 4.94	8.61 ± 4.79	0.041	1.145 (0.951–1.378)	0.154	1.209 (0.991–1.475)	0.061
Aneurysm wall enhancement	9 (75.0%)	17 (41.5%)	0.042	4.235 (0.997–17.999)	0.051	6.566 (1.395–30.903)	0.017
Thrombosis in aneurysm sac	3 (25.0%)	12 (29.3%)	1.000	1.067 (0.241–4.715)	0.932		
Patent artery stenosis	2 (16.7%)	8 (20.0%)	1.000	0.800 (0.145–4.399)	0.797		
Left aneurysm side	4 (33.3%)	12 (29.3%)	1.000	1.208 (0.305–3.783)	0.787		
Aneurysm morphology			1.000	0.841 (0.085–8.323)	0.882		
Saccular	11 (91.7%)	37 (90.2%)					
Fusiform	1 (8.3%)	4 (9.8%)					
Adjunctive coiling	2 (16.7%)	5 (12.2%)	0.650	1.440 (0.242–8.567)	0.689		
Jailed branch	11 (91.7%)	36 (87.8%)	1	0.655 (0.069–6.215)	0.712		
Any jailed M2 trunk	9 (75.0%)	20 (48.8%)	0.187	0.317 (0.075–1.344)	0.119		
Alloy of FDs			0.207	0.343 (0.078–1.506)	0.156		
Nickel-Titanium	4 (33.3%)	6 (14.6%)					
Cobalt Chromium	8 (66.7%)	35 (85.4%)					

OR, odds ratio; CI, confidence interval.

## Discussion

Traditionally, MCA aneurysms can be managed by either microsurgical clipping or endovascular coiling with or without stent assistance. A multicenter retrospective study comparing microsurgical clipping and endovascular treatment for MCA aneurysms demonstrated that clipping achieved higher aneurysm occlusion rates and lower retreatment rates, while no significant differences in clinical outcomes were observed between the two treatment strategies (7). The application of FDs in the treatment of MCA aneurysms has been investigated in several studies (M2-M3) (8–11). These studies mainly focused on aneurysms in the distal circle of Willis, including M2, M3; conclusive evidence supporting the use of FDs in M1 aneurysms is still emerging. In our study, we reported that the major ischemic complications were 25.9% and the overall complete aneurysm occlusion of MCA M1 aneurysms with FDs was 77.4%. Although these findings demonstrate a relatively high rate of occlusion with the use of FDs, the risk of procedure-related ischemic complications cannot be ignored.

At the final clinical follow-up of our study, 96% of patients achieved favorable clinical outcomes at the final follow-up. However, 15 patients experienced procedure-related ischemic complications, among whom three developed long-term neurological deficits (mRS = 4). Fortunately, most patients with complications had favorable outcomes without lasting neurological impairment. Of note, all patients with ischemic complications had side branches covered by the FDs; however, no significant correlation was observed between FDs-covered branches and ischemic events. This finding is consistent with previous reports that Diestro et al. (12) reported that 85.2% of MCA aneurysms treated with FDs involved jailed branches, but no association was found between jailed branches and thromboembolic complications. It is plausible that collateral arterial supply contributes to maintaining sufficient perfusion in covered branches, mitigating ischemic risk (10).

Notably, ruptured aneurysms were identified as a significant risk factor for postoperative ischemic events in our cohort treated with FDs for MCA M1 segment aneurysms (OR, 15.273; 95% CI, 1.547–150.765; *p* = 0.020). This observation aligns with one previous study (12). Several mechanisms may account for this association. First, subarachnoid hemorrhage following aneurysmal rupture may induce severe cerebral vasospasm, thereby predisposing to ischemic injury (13). Second, antiplatelet preparation is often suboptimal in the acute phase of ruptured aneurysms, which may increase the risk of thromboembolic complications after FDs deployment. A recent multicenter retrospective study from 10 high-volume cerebrovascular centers in the United States compared microsurgical clipping, simple coiling, stent-assisted coiling, and flow diversion for the treatment of MCA bifurcation aneurysms. The authors reported that the use of flow diverters was associated with higher rates of vasospasm and thromboembolic complications compared with other treatment strategies (14). These findings are consistent with those observed in our study.

To date, no standardized antiplatelet regimen has been established for the use of FDs in ruptured aneurysms. Based on our clinical experience, dual antiplatelet therapy is initiated 2–6 h before emergent endovascular treatment in patients requiring intracranial stent or FDs implantation. During the procedure, a weight-adjusted intravenous bolus of tirofiban is administered immediately before FDs deployment, followed by a 24-h continuous infusion. Tirofiban possesses a short half-life (2–4 h) and a rapidly reversible antiplatelet effect within approximately 3 h after discontinuation, offering a favorable safety profile in the acute setting. A previous study involving 60 patients with ruptured intracranial aneurysms treated with FDs reported a postoperative stroke rate of 10% when tirofiban was used during periods of insufficient antiplatelet coverage (15), which was notably lower than that in our series. Notably, the ischemic complication rate among ruptured aneurysms in our cohort was particularly high. Although the sample size was limited, this findings highlights the potential risks associated with FDs treatment in the acute rupture setting. Therefore, the use of flow diversion in acutely ruptured aneurysms should be approached with extreme caution and should generally be avoided unless no other treatment options, such as coiling, stent-assisted coiling, or microsurgical clipping, are feasible.

Previous studies support platelet function–guided antiplatelet strategies in endovascular treatment of intracranial aneurysms. A randomized trial showed that replacing clopidogrel with ticagrelor in patients identified by light transmission aggregometry (LTA) significantly reduced ischemic events after treatment of unruptured aneurysms (16). Similarly, a recent multicenter study demonstrated that platelet function test–guided antiplatelet therapy was associated with fewer postoperative ischemic complications (17). However, ruptured aneurysms were not specifically evaluated in these studies. Given the distinct pathophysiological features of ruptured intracranial aneurysms, whether platelet function–guided antiplatelet management can reduce ischemic complications in this population remains to be determined.

The effects of FDs on MCA branch vessels have been a subject of ongoing concern. In our study, we specifically examined ischemic event rates in relation to coverage of major M2 branches by flow diverters; however, no significant association was identified between M2 branch coverage and postoperative cerebral ischemia. These findings are consistent with emerging evidence suggesting that branch coverage alone does not necessarily translate into clinically relevant ischemic injury. A recent single-center retrospective study further evaluated branch involvement and hemodynamic alterations following flow diverter treatment of MCA aneurysms and assessed their relationship with postoperative DWI lesions and neurological deficits. The authors reported that although flow diversion frequently resulted in changes in branch flow and/or vessel caliber, most patients remained clinically asymptomatic. Moreover, while DWI-detected ischemic lesions were commonly observed after treatment, only a minority of cases developed corresponding neurological symptoms (18). Taken together, these observations suggest that cerebral infarction following flow diversion in the setting of MCA branch coverage is likely multifactorial, and the precise mechanisms linking branch involvement to clinically significant ischemia remain to be further elucidated.

In our cohort, no patient had hemorrhagic complications. However, the risk of intracerebral hemorrhage cannot be ignored. Zhu et al. (6) reported a 3.2% rate of hemorrhagic complication in MCA aneurysms treated with Pipeline, and a meta-analysis showed a 2.9% overall rate for FDs (19). As some FDs systems are stiff, a high-procedure technique is needed, especially for distal aneurysms with smaller vessel lumens (10). In addition, considering that ischemic complications were more frequent in our cohort, hemorrhagic transformation after infarction should be considered, especially in patients who were under dual anti-platelet management after the procedure (20).

Our results indicate that AWE observed prior to FDs implantation is an independent risk factor for aneurysm recurrence (odds ratio: 6.566, 95% confidence interval: 1.395–30.903, *p* = 0.017). AWE is generally considered a marker of inflammatory cell infiltration and the release of proinflammatory cytokines within the aneurysm wall, suggesting structural instability (21–23). Therefore, VWI has been increasingly used to assess the rupture risk of aneurysms. Growing evidence suggests that AWE may be associated with treatment outcomes. In a prospective, single-center cohort study conducted at our institution, VWI was used to predict outcomes following the surgical clipping of intracranial aneurysms. The study found that the pattern of wall enhancement was associated with both surgical outcomes and the risk of complications (24). Another study investigating the relationship between AWE and endovascular treatment of unruptured aneurysms demonstrated that circumferential enhancement significantly increased the risk of aneurysm recurrence (OR: 14.2, 95% CI: 1.8–110.8, *p* = 0.01). Pre-treatment AWE suggests ongoing inflammation within the aneurysm wall, which may persist even after FDs placement and potentially interfere with endothelization, thereby impairing aneurysm healing. Neointima formation is crucial for the success of endovascular therapy for intracranial aneurysms after FDs implantation (25). Prolonged inflammation in the aneurysm wall before endothelization can lead to wall remodeling, increased wall fragility, inadequate thrombus organization, and ultimately, failed neointima formation (26). Although FDs provide a scaffold for endothelial cell migration across the aneurysm neck, inflammation within the aneurysm wall may hinder this. Therefore, further prospective studies are warranted to clarify the role of AWE in predicting treatment outcomes and guiding therapy selection.

Furthermore, our results indicated that smaller intracranial aneurysms tended to have a higher likelihood of incomplete occlusion following FDs implantation (OR 1.209; 95% CI 0.991–1.475, *p* = 0.061). Although previous studies have generally suggested that smaller aneurysms are more likely to achieve complete occlusion (27, 28), these studies typically used a 10 mm threshold to define aneurysm size categories. In contrast, we did not dichotomize aneurysm size based on this conventional cutoff. Moreover, a recent meta-analysis reported that aneurysm size may not be a significant determinant of occlusion outcomes following FDs treatment (29).

At our center, adjunctive coiling is routinely performed for large or giant aneurysms prior to FDs deployment. This approach is intended to reduce the hemodynamic impact of continued flow against the aneurysm wall after FDs placement and to facilitate earlier thrombosis and healing. In our cohort, adjunctive coiling was not significantly associated with complete aneurysm occlusion. However, previous studies have shown that for large aneurysms or those at a higher risk of rupture, coil-assisted embolization may enhance occlusion rates and reduce the need for retreatment (30). Given that the decision to use adjunctive coiling is often based on aneurysm size and morphology, the potential for confounding factors exists. Therefore, further research is needed to determine the optimal aneurysm size threshold at which adjunctive coiling should be employed to reduce the risk of incomplete occlusion following FDs treatment.

This study compared FDs made of nickel–titanium (NiTi) and cobalt–chromium (CoCr) alloys regarding ischemic complications and arterial occlusion. Although not statistically significant, NiTi FDs showed fewer ischemic complications but a higher rate of incomplete occlusion. These differences may relate to alloy properties, as the greater flexibility of NiTi devices could reduce wall apposition compared with stiffer CoCr devices, potentially affecting efficacy. Given the limited sample size, these findings require validation in larger studies.

We acknowledge several limitations. First, the retrospective single-center design inherently limits the strength and generalizability of the findings and may introduce selection bias, while outcome assessment performed by the investigators may also be subject to potential bias. Second, the sample size was relatively small, reflecting the limited use of flow diverters for MCA aneurysms in routine clinical practice. Third, although multiple flow diverter devices available at our institution were included, the number of cases for some devices was insufficient to permit meaningful comparisons of efficacy among different stent platforms. Therefore, larger prospective studies are required to provide more comprehensive insights.

## Conclusion

Flow diversion for MCA M1 segment aneurysms was associated with high occlusion rates and favorable clinical outcomes. However, ruptured aneurysms were linked to a markedly increased risk of postoperative ischemic complications; therefore, FDs should be used with caution in the acute rupture setting. In addition, aneurysm wall enhancement on preoperative MRI independently predicted incomplete aneurysm occlusion after FDs treatment.

## Data Availability

The original contributions presented in the study are included in the article/supplementary material, further inquiries can be directed to the corresponding authors.
